# Emerging *Neisseria meningitidis* ST-1466 and ST-11026 as urogenital pathogens in China

**DOI:** 10.1186/s12879-025-11907-1

**Published:** 2025-10-29

**Authors:** Zhe Zhu, Youwei Bao, Ziyan Zhang, Lina Ye, Yefang Ke

**Affiliations:** 1 Department of Blood Transfusion, Ningbo No.2 Hospital, Ningbo, Zhejiang China; 2 The Central Laboratory of Birth Defects Prevention and Control, Women and Children’s Hospital of Ningbo University, Ningbo, Zhejiang China; 3 Key Laboratory of Systems Health Science of Zhejiang Province, School of Life Sciences, Hangzhou Institute for Advanced Study, University of Chinese Academy of Sciences, Hangzhou, Zhejiang China; 4 Department of Clinical Laboratory, Women and Children’s Hospital of Ningbo University, Liuting Street 339, Ningbo, People’s Republic of China; 5 Ningbo Key Laboratory for the Prevention and Treatment of Embryogenic Diseases, Women and Children’s Hospital of Ningbo University, Ningbo, China

**Keywords:** *Neisseria meningitidis*, Urogenital pathogen, Meningococcal urethritis, China, Infertility

## Abstract

**Background:**

*Neisseria meningitidis*, known primarily for causing severe sepsis and meningitis, has also been identified as a cause of urethritis. However, reports of such cases in China are scarce. We present two cases of *N. meningitidis* strains isolated from the urogenital tract in China in 2024.

**Methods:**

Two *N. meningitidis* isolates were recovered from the urogenital tract. Whole genome sequencing (WGS) was performed to identify genogroup and sequence type (ST), factor H binding protein (fHbp), capsule polysaccharide synthesis (*cps*) and nitrite reductase (*aniA*) gene. Phylogenetic analysis was also performed.

**Results:**

The first case was an infertile female with significant symptoms of urethritis. The strain isolated from her cervical secretion (FE-07) was identified as genogroup Y, ST-1466, clone complex (cc)-174, fHbp variants 2.21, harboring intact *cps* locus, and frameshift-mutated *aniA* gene. The second case was an infertile male presenting with mild pruritus of the glans. The strain recovered from his semen (FE-08) was classified as nongroupable, ST-11026, cc-32, fHbp variants 1.1, harboring incomplete *cps* locus, and frameshift-mutated *aniA* gene. Both STs of *N. meningitidis* have not been reported in China previously and are phylogenetically distinct from strains with the same STs in the NCBI GenBank database.

**Conclusions:**

Our study highlights the emergence of *N. meningitidis* as a urogenital pathogen in China and underscores the need for vigilance against potential outbreaks of meningococcal urethritis.

**Supplementary Information:**

The online version contains supplementary material available at 10.1186/s12879-025-11907-1.

## Introduction

*Neisseria meningitidis*, a Gram-negative coccus commonly colonizing the nasopharynx of humans, is a well-known cause of severe invasive meningococcal disease (IMD), primarily manifests as meningococcal sepsis and meningitis [1]. The incidence of IMD was low worldwide, but it varied significantly across different regions, with relatively high in Africa. Serogroups A, B, C, W and Y accounted for the most IMD cases, mainly affects infants and young children [2]. Although *N. meningitidis* and *Neisseria gonorrhoeae* originated from a common ancestor, they are distinct pathogens causing different diseases, with the latter primarily associated with urogenital infections and sexual transmission [3, 4]. However, sporadic cases of meningococcal urethritis have been reported with symptoms similar to gonococcal urethritis, indicating that *N. meningitidis* can also cause urogenital infections [3].

The isolation of *N. meningitidis* from the urogenital tract can be traced back to the early twentieth century but started to increase in the 1970 s, parallel to the HIV pandemic, particularly among men who have sex with men (MSM) [3–5]. Since 2015, an outbreak of meningococcal urethritis caused by the United States *N. meningitidis* urethritis clade (US_-_NmUC) occurred in the US, primarily among heterosexual males [6–8]. The US_-_NmUC isolates belong to sequence type (ST)−11, clonal complex (cc) 11, and this strain was subsequently reported in the United Kingdom (UK), Vietnam, and Japan [9–11], suggesting it continues to circulate. Oral sex is considered a likely transmission route for urethral meningococcal infection [3].

To date, only one case of meningococcal urethritis has been reported in China. This involved an HIV-seropositive man in Lishui who engaged in oral sex with female partners, from whose urethral specimen *N. meningitidis* was isolated, exhibiting a novel ST (unassigned in PubMLST) [12]. In this study, we described two cases with *N. meningitidis* isolated from cervical secretion and semen, respectively, in Ningbo, China in 2024.

## Methods

### N. meningitidis isolates collection

*N. meningitidis* isolates (FE07, and FE08) were recovered from the urogenital tract of two patients with infertility, admitted to the Women and Children’s Hospital of Ningbo University for in vitro fertilization (IVF). Prior to IVF, genital tract samples were collected for *N. gonorrhoeae* testing as part of routine medical examination. Briefly, cervical secretion from the female patient and semen from the male patient were collected, then plated on Chocolate agar + PolyViteX VCAT3, and incubated at 35℃ in 5% CO_2_. Suspicious isolates were identified by matrix-assisted laser desorption ionization-time of flight mass spectrometry (MALDI-TOF MS) (BioMérieux, France).

Antimicrobial susceptibility testing (ASTs) was performed using the disk diffusion method, following Clinical and Laboratory Standards Institute (CLSI) guidelines [13]. Antimicrobial agents tested included cefotaxime (30 μg), ceftriaxone (30 μg), meropenem (10 μg), ciprofloxacin (5 μg), chloramphenicol (30 μg), and trimethoprim-sulfamethoxazole (TMP-SMX) (1.25/23.75 μg).

### Whole genome sequencing (WGS), and phylogenetic analysis

Genomic DNA was extracted using the TIANamp Bacteria DNA kit (Tiangen Biotech, China). Whole genome sequences were generated using a hybrid assembly approach with Unicycler (v0.4.5) [14], combining long-read data from the Nanopore MinION Platform (Nanopore, Oxford, UK) and short-read data from the Illumina NovaSeq 6000 Platform (Illumina Inc, San Diego, U.S.A). ST, finetyping, antimicrobial resistance genes (ARGs), capsule polysaccharide synthesis (*cps*) genes, and factor H binding protein (fHbp) variants were determined using tools available on the *Neisseria* PubMLST website (https://pubmlst.org/). WGS-based genogrouping was conducted following previously reported methods [15, 16]. The genetic context of the *cps* locus was analyzed using BLASTn v2.4.0, and visualized using Easyfig. For the nitric oxide reductase-nitrite reductase (*norB-aniA*) gene cassette, we first compared aniA protein sequences between FE-07, FE-08, *N. meningitidis* strain FAM 18 (Accession: AM421808), *N. gonorrhoeae* strain FA19 (Accession: CP012026), and US_-_NmUC strain COL-201504–11 (Accession: NZ_CP017257) to identify *aniA* frameshift mutation by using ESPript 3.0 software [17], then conducted a phylogenetic analysis of *norB-aniA* gene sequence for these isolates using MEGA-X software.

We performed phylogenetic analyses on isolates FE-07 (ST-1466) and FE-08 (ST-11026) using whole-genome sequences of *N. meningitidis* retrieved from the GenBank database (https://www.ncbi.nlm.nih.gov/genbank/) (accessed October 7, 2024). Specifically, isolates identified as ST-1466 and ST-11026 were selected and underwent stringent quality control, requiring a genome size of 1.9–2.2 Mb, GC content of 51–53%, and a contig count ≤ 500. Core-genome single nucleotide polymorphism (SNP)-based phylogenetic trees were constructed using the Harvest suite. Parsn was used to calculate pairwise SNPs after removing genomic sequences in the recombination regions. Maximum-likelihood phylogenetic trees with 1,000 bootstraps were generated using IQ-TREE v1.6.1. The phylogenetic trees and associated data were visualized with iTOL v7 (10.1093/nar/gkab301).

### Clinical data collection and ethics approval

Clinical data were collected from electronic medical records, and informed consent was obtained from both patients in this study. The ethics committee of Women and Children’s Hospital of Ningbo University approved this study (EC2024-187).

## Results

### Clinical characteristics of the patients

The FE07 isolate was recovered from the cervical secretion of a 37-year-old infertile female (Case 1) on July 24, 2024 (Table 1). She had a history of two abortions eight years prior, followed by intrauterine adhesion. Despite four hysteroscopic surgeries, the adhesions persisted, prompting her to seek IVF treatment. Two days before this pre-IVF routine examination, she had symptoms of frequent urination and urgency, with a urinary white blood cell (WBC) count of 13.41 per high-powered field (hpf).

**Table 1:** The information of two *N*. *meningitidis* strains isolated from the urogenital tract in China in 2024

Isolate	Date	Host gender	Host age	Sample type	Sequence Type	Clonal complex	Genogroup	fHbp variant	Fine type	
									PorA	FetA
FE07	2024	Female	37 years	Cervical secretion	1466	174	Y	2.21	21,16	F3-7
FE08	2024	Male	28 years	Semen	11,026	32	Nongenogroupable	1.1	5–2,13–15	F5-1

*fHbp* Factor H binding protein

The FE08 isolate came from the semen of a 28-year-old infertile male (Case 2) on August 19, 2024 (Table 1). He presented with mild itching of the external urethral orifice.

Both patients were heterosexual, and reported one sex partner each (their spouse), and denied engaging in oral sex. No *Ureaplasma urealyticum*, *Mycoplasma hominis*, *Chlamydia trachomatis*, *Trichomonas vaginalis*, HIV or syphilis was detected in the patients.

Both patients were treated with oral cefuroxime 250 mg bid. The urinary tract infection symptoms resolved within one week. *N. meningitidis* was not isolated from the cervical secretion of the female patient one week later, nor recovered from the semen of the male patient two months later during follow-up for continued IVF treatment.

### Information on N. meningitidis isolates

The FE07 isolate was susceptible to all tested antimicrobial agents (Table 2). WGS identified the FE07 isolate as genogroup Y, ST-1466, clone complex (cc)−174, with a fine type of PorA 21, 16; FetA F3-7 (Table 1).

**Table 2:** Antimicrobial susceptibility tests of two *N. meningitidis* strains isolated from the human urogenital tract in China in 2024

Antimicrobial agent	FE07		FE08		Breakpoints		
	Zone Diameter (mm)	Interpretation	Zone Diameter (mm)	Interpretation	S (mm)	I (mm)	R (mm)
Cefotaxime (30 μg)	44	S	40	S	≥ 34	-	-
Ceftriaxone (30 μg)	44	S	44	S	≥ 34	-	-
Meropenem (10 μg)	33	S	31	S	≥ 30	-	-
Ciprofloxacin (5 μg)	40	S	30	R	≥ 35	33–34	≤ 32
Chloramphenicol (30 μg)	35	S	30	S	≥ 26	20–25	≤ 19
TMP-SMX (1.25/23.75 μg)	33	S	6	R	≥ 30	26–29	≤ 25

*TMP-SMX* Trimethoprim-sulfamethoxazole

FE08 was susceptible to cefotaxime, ceftriaxone, meropenem, and chloramphenicol but resistant to ciprofloxacin, and TMP-SMX (Table 2). WGS classified the isolate as non-genogroupable, ST-11026, cc-32, with a fine type of PorA 5–2, 13–15; FetA F5-1 (Table 1). In addition, the *gyrA* T91I mutation was identified, which is known to confer resistance to ciprofloxacin.

**Fig. 1:**
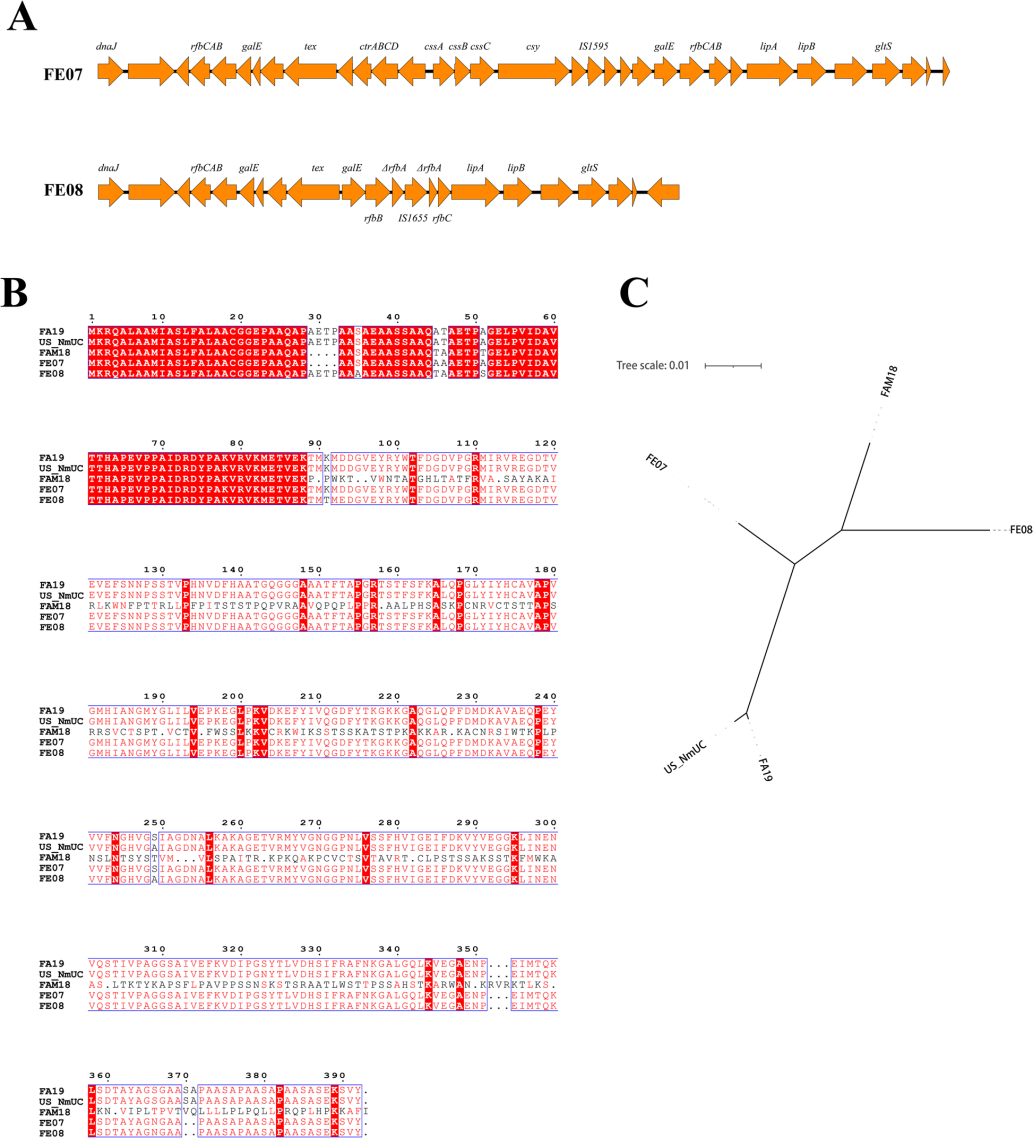
Characteristics of *cps* locus and *norB-aniA* gene cassette in two *N*. *meningitidis* strains (FE-07 and FE-08) isolated from the human urogenital tract in China in 2024. **A** Genetic organization of the *cps* locus in FE-07 and FE-08. **B** AniA protein sequence alignment among FE-07, FE-08, FA19 (*N. gonorrhoeae* strain), US_-_NmUC, and FAM 18 (*N. meningitidis* strain), using the full-length FA19 AniA as reference. Identical matches are represented by red background with white text, high similarity by red text, and low similarity by black text. **C** Phylogenetic tree of *norB-aniA* gene sequences of FE-07, FE-08, FA19, US_-_NmUC, and FAM 18

The *cps* locus was intact in isolate FE-07, whereas FE-08 exhibited an incomplete *cps* locus—lacking both capsule synthesis genes and capsule transport genes (Fig. 1A). Regarding fHbp, FE-07 and FE-08 isolates harbored variants 2.21 and 1.1, respectively (Table 1). The aniA protein sequence of FE-07 and FE-08 had amino acid lengths of 383, and 387, respectively, shorter than gonococcal and US_-_NmUC isolates (392) (Fig. 1B), exhibiting frameshift mutations in the *aniA* gene. Phylogenetic analysis of the *norB-aniA* cassette revealed that FE-07 and FE-08 did not cluster with gonococcal or US_-_NmUC isolates (Fig. 1C).

### Phylogenetic analysis

As of October 7, 2024, 9,525 *N. meningitidis* genomes were downloaded from GenBank, among which 357 originated from China. Of these 9,525 isolates, 96 were ST-1466 and 25 were ST-11026. One ST-1466 genome failed sequence data quality control and was excluded, resulting in the final selection of 95 ST-1466 and 25 ST-11026 genomes. This final set of isolates were used for comparison with FE-07 and FE-08, respectively (Supplementary materials).

**Fig. 2:**
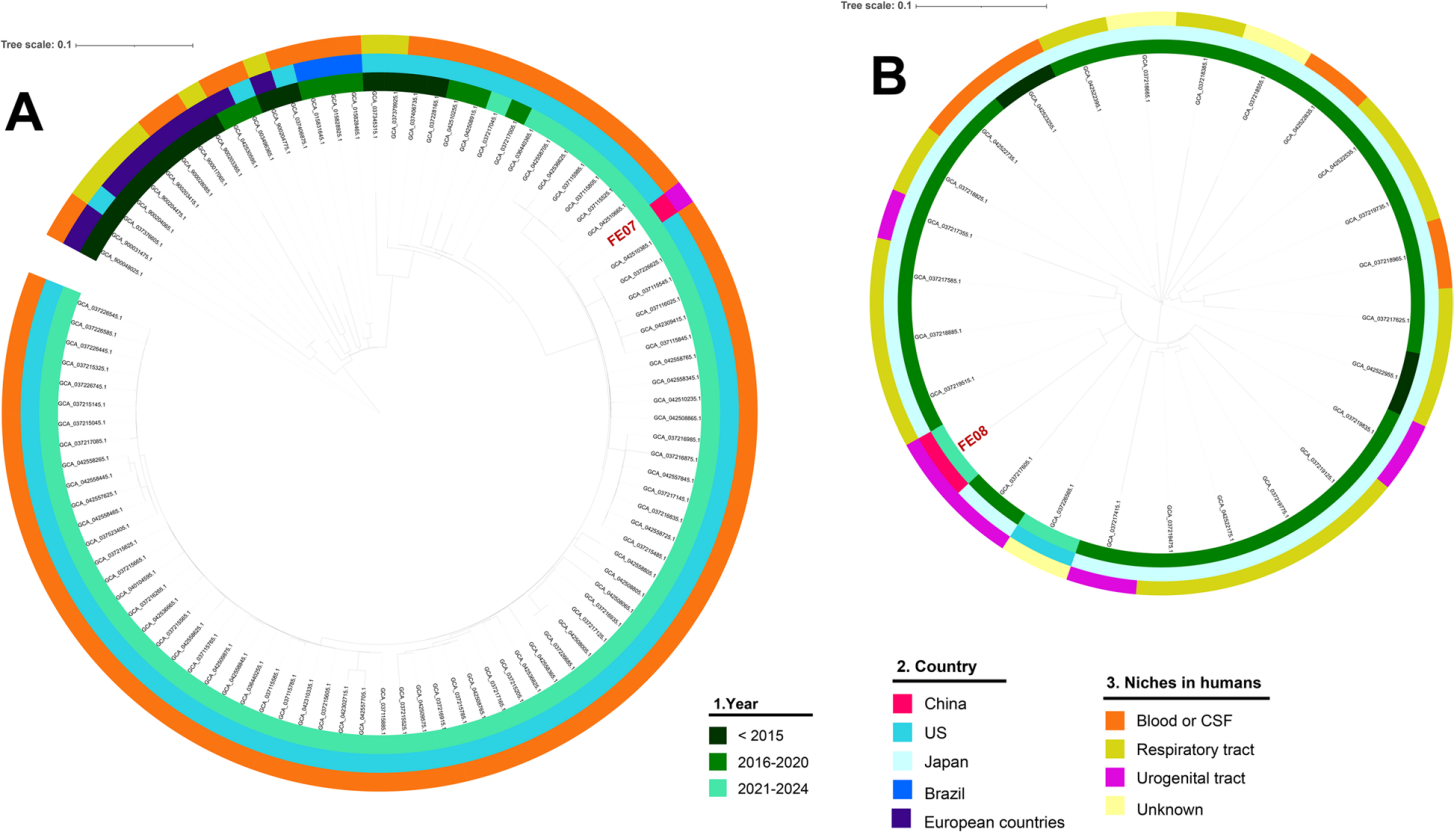
Phylogenetic analysis of two *N*. *meningitidis* strains (FE-07 and FE-08) isolated from the human urogenital tract in China in 2024. The rings from the inner to outer indicate year of isolation, country of origin, and anatomical site of isolation. **A** The phylogenetic tree with FE-07 and 95 *N. m*eningitidis ST-1466 genomes from NCBI GenBank uses *N. m*eningitidis ST-1466 (GCA_900031475.1) as a reference genome. **B** The phylogenetic tree with FE-08 and 25 *N. m*eningitidis ST-11026 genomes from NCBI GenBank uses *N. m*eningitidis ST-11026 (GCA_042522735.1) as a reference genome

Most *N. meningitidis* ST-1466 genomes in the NCBI GenBank database were from the US (82/95, 86.3%), the others were from Brazil, the UK, the Netherlands, and Belgium, and none were from China. The isolates were primarily recovered from blood or CSF samples (87/95, 91.58%), with the rest from nasopharyngeal swabs (8/95, 8.42%), and none from the urogenital tract. Phylogenetic analysis based on core-genome SNP analysis revealed that the FE07 isolate did not cluster with other *N. meningitidis* ST1466 genomes in the NCBI GenBank database (Fig. 2A). The closest strain was a 2023 US CSF isolate (GCA_042558765.1), differing by 87 SNPs.

As for the genetic relatedness of FE08 isolate (ST-11026, cc-32), we selected *N. meningitidis* ST-11026 genomes (n = 25) from the NCBI GenBank database for phylogenetic analysis. The majority (24/25) of *N. meningitidis* ST-11026 genomes were from Japan, isolated during 2014–2020, with one from the US in 2023. Among the 25 ST-11026 strains, fourteen were from respiratory tract specimens (56%), four from the urogenital tract (16%), three from blood (12%), one from synovial fluid (4%), the other three from unknown sources (12%). The FE08 isolate did not cluster with other ST-11026 strains in the NCBI GenBank database. The core-genome SNP differences generally exceeded 1000, with the closest strain showing 940 differences (GCA_037219515.1, from a sputum sample in Japan, 2017) (Fig. 2B).

## Discussion

In this study, we isolated *N. meningitidis* from the urogenital tract in two infertile patients with significant or mild symptoms of urethritis. The *N. meningitidis* strains were identified as ST-1466 (cc-174) and ST-11026 (cc-32), respectively. Both STs of *N. meningitidis* have not been reported in China previously and are phylogenetically distinct from strains with the same STs in the NCBI GenBank database.

Although sporadic, urogenital infections by *N. meningitidis* are on the rise. Especially, there are two notable points worth paying attention to. First, a significant cluster of meningococcal urethritis cases emerged in the US in 2015 [6–8], and spread to the UK, Vietnam and Japan [9–11], linked to the US_-_NmUC isolates, a nongroupable clonal *N. meningitidis* ST-11, cc11. These isolates have adapted to the urogenital niche by losing the capsule, expressing a hybrid *fHbp* gene, and acquiring the gonococcal *norB-aniA* gene cassette, suggesting *N. meningitidis* may develop mechanisms to thrive in the urogenital environment [4]. Second, the first reported case of meningococcal urethritis in China involved an HIV-positive individual [12], indicating the emergence of urethritis-associated *N. meningitidis* in China.

Genomic features potentially linked to urogenital infection in US_-_NmUC isolates were assessed in strains FE-07 and FE-08. First, the *cps* locus was intact in isolate FE-07, suggesting the capsule was not involved in its urogenital colonization. Conversely, FE-08 exhibited an incomplete *cps* locus, consistent with Japanese ST-11026 meningococcal isolates [18]. Although involving distinct mechanisms, capsule loss in FE-08 may facilitate urogenital tract adaptation, similar to that observed in US_-_NmUC strains. Second, FE-07 and FE-08 harbored fHbp variants 2.21 and 1.1, respectively. Variant 1 strains have been shown to express significantly higher levels of fHbp compared to variant 2 and variant 3 strains [19]. Consequently, fHbp expression in FE-07 (variant 2.21) may not contribute to urogenital pathogenesis, whereas the highly expressed variant 1.1 fHbp in FE-08 likely promotes urogenital tropism. Third, the *norB-aniA* cassette sequences of FE-07 and FE-08 were phylogenetically distinct from gonococcal and US_-_NmUC isolates and contained frameshift mutations in *aniA*. This suggests the *norB-aniA* cassette is likely non-functional in these isolates and is unlikely to contribute to urogenital infection.

Transmission of *N. meningitidis* from the upper respiratory tract to the urogenital tract via oral sex is a plausible route [4, 5]. However, unlike the first Chinese case and other meningococcus urethritis cases involving oral sex [12, 20, 21], the patients in this study denied engaging in oral sex. Unfortunately, the upper respiratory secretions of their partners were not collected for *N. meningitidis* identification, leaving the origin of the strains uncertain. Besides, different from the first Chinese case which had a novel sequence type (unassigned in PubMLST) [12], and the prevalent ST-11 urethrotropic clade [6–8], as well as other urogenital *N. meningitidis* STs (e.g., ST-32, ST-4821, ST-23) [22, 23], the isolates in this study were ST-1466 and ST-11026, highlighting the diverse STs of urogenital *N. meningitidis* isolates.

*N*. *meningitidis* serogroup Y ST-1466 was recently identified as a pathogen responsible for (IMD) with a high case-fatality rate. During 2022–2024, *N. meningitidis* serogroup Y ST-1466 caused an IMD outbreak in Virginia, US, with 36 cases and 7 deaths (19.4%) [24]. Furthermore, it has become one of the leading IMD strains in the US, with serogroup Y responsible for most cases, and ST-1466 accounting for 68% (101/148) of serogroup Y cases in 2023 (https://www.cdc.gov/han/2024/han00505.html). Concurrently, an outbreak of *N. meningitidis* serogroup Y ST-1466 caused 41 urogenital infections in Australia [25]. These outbreaks underscore that *N*. *meningitidis* serogroup Y ST-1466 can cause both IMD epidemics and large-scale urogenital infections. In this study, an *N. meningitidis* ST-1466 isolate (FE07) was recovered from the cervical secretion of a female with urinary tract infection symptoms. Although as far as we know *N. meningitidis* Y ST-1466 has not been previously reported in China, vigilance is warranted for potential IMD and urethritis epidemics caused by this strain.

*N*. *meningitidis* ST-11026 was unique to Japan previously [18, 26]. A total of 24 ST-11026 strains were isolated from respiratory specimens, blood, or urogenital tract in Japan during 2014–2020 [18], indicating that *N*. *meningitidis* ST-11026 can colonize, cause invasive disease, and be sexually transmitted. In the Japanese study, although 4 urogenital strains cluster with other 6 stains from respiratory specimen cluster together to form a subcluster, all 24 strains appeared to be genetically very closely within ST-11026 meningococci [18]. To our knowledge, *N*. *meningitidis* ST-11026 has not been reported in China before. Interestingly, the *N*. *meningitidis* ST-11026 strain (FE08) in this study was ciprofloxacin resistant, consistent with most Japanese ST-11026 isolates (75%, 18/24), due to a *gyrA* T91I mutation [25]. Ciprofloxacin is used for chemoprophylaxis in close contacts of IMD patients, and the *gyrA* T91I mutation plays a key role in ciprofloxacin resistance among* N*. *meningitidis* isolates [26, 27]. Other ciprofloxacin-resistant strains in China, such as serogroup A cc5 and serogroup C/B cc4821, also exhibit the *gyrA* T91I mutation [27]. With the emergence of new STs of *N*. *meningitidis* with ciprofloxacin resistance, chemoprophylaxis by ciprofloxacin would raise more concerns.

We tried to assess the origin and evolution of the FE07 and FE08 isolates by using phylogenetic analysis based on the core genomes. However, the FE07 and FE08 isolates were phylogenetically unrelated to other isolates with the same STs, indicating unknown origins. It should be noted that out of the 95 strains of ST-1466 in NCBI GenBank database, only 10 were from European countries, while the PubMLST database lists more than 200 isolates of ST-1466 from European countries.

Interestingly, these two *N*. *meningitidis* strains were both isolated from patients with infertility. By scanning the literature, there was a 28-year-old infertile man with *N*. *meningitidis* ST23 serogroup Y isolated from the semen, however, there was no obvious association with infertility [23]. Studies have revealed that *N. gonorrhoeae* can induce male and female infertility [28, 29]. The relationship between urogenital *N*. *meningitidis* infection and infertility remains uncertain and requires further investigation.

The prevalence of *N*. *meningitidis* isolates in the urogenital tract may be underestimated, as routine methods using culture on selective medium, Gram stain, oxidase reaction, and carbohydrate fermentation may misidentify meningococcus as gonococcus [4]. MALDI-TOF MS is a useful tool to discriminate *N. gonorrhoeae* and *N*. *meningitidis* isolates [4]. The *N*. *meningitidis* isolates in this study were identified by MALDI-TOF MS, which has been employed in our laboratory since 2023, while no *N*. *meningitidis* strain was detected from the urogenital tract before 2023 by using routine methods. This indicates the urogenital *N*. *meningitidis* strain is an emerging pathogen, or may have been missed using previous methods. Since the widespread use of MALDI-TOF MS in clinical laboratories, urogenital* N*. *meningitidis* strains may be detected more frequently.

There are no recommendations for urogenital meningococcal infection treatment currently, some studies used the treatments for gonococcal disease [3]. The treatments were similar across different countries: cephalosporin with or without azithromycin, which showed effective eradication of the pathogen [12, 20, 21, 30]. Both cases in our study were successfully treated with oral cefuroxime for presumptive gonorrhea. The urinary tract infection symptoms resolved, and *N. meningitidis* was not isolated from their urogenital tracts during follow-up.

## Conclusions

In conclusion, this study reports two cases of urethral meningococcal infection caused by two emerging *N*. *meningitidis* STs in China. Health Regulatory Authorities and clinicians should consider *N*. *meningitidis* as a urogenital pathogen as part of the differential diagnosis, and remain vigilant against potential outbreaks of meningococcal urethritis.

## Supplementary Information

Supplementary Material 1.

## Data Availability

The complete nucleotide sequences of the FE07 and FE08 isolates were deposited to GenBank of NCBI under the accession number PRJNA1195405 (https://www.ncbi.nlm.nih.gov/bioproject/PRJNA1195405).

## Abbreviations

WGS: Whole genome sequencing
ST: Sequence type
cc: Clonal complex
MSM: Men who have sex with men
US_-_NmUC: United States *N. meningitidis* urethritis clade
UK: United Kingdom
IVF: In vitro fertilization
MALDI-TOF MS: Matrix-assisted laser desorption ionization-time of flight mass spectrometry
AST: Antimicrobial susceptibility test
CLSI: Clinical and Laboratory Standards Institute
WBC: White blood cell
